# Supplementary material for: Ebola incidence and mortality before and during a lockdown: The 2022 epidemic in Uganda
Source: PLOS Glob Public Health. 2023 Dec 22;3(12):e0002702. doi: 10.1371/journal.pgph.0002702 (PMC10745136; doi:10.1371/journal.pgph.0002702)
Supplement: Attachment — Submitted filename: Reviews for Izumi J et al.docx [file pgph.0002702.s003.docx]

In the article titled ‘Ebola incidence and mortality before and during a lockdown: the 2022 epidemic in Uganda’, Izudi J et al assess the incidence of cases and mortality peri-lockdown during the 2022 SUDV outbreak in Uganda. I found the paper to be generally well written and the methods appropriate.

I, however, hold the two concerns below:

Major:

- The authors need to inform the Uganda MOH leadership about their utilization of the data in this manner; either via the Director General or Strategic Management committee. This should easily be done by sharing a summary of the write up of results or asking for a chance to present these findings as a dissemination activity. When completed, that dissemination activity can then be noted in the paper. This will override the social controversy of use of public epidemic data in absence of awareness or permission from the owner. While it’s not unethical; this data surrounds human subjects and we need to show social responsibility to the authorities and responders.

Minor

- The conclusion should be restricted to inferences from the results. Any other insights should clearly be given as recommendations.
- As it stands now, for example, the abstracts conclusion is extended to a recommendation that is not inferred from the results of the study.
- I argue that any recommendation should be taken out of the the abstract and left for main body of the manuscript to avoid confusion
- Conclusion ‘Overall, the EVD incidence and mortality remained higher in the early weeks of the lockdown than during the pre-lockdown period nationally and in one of the two districts. With extended lockdown, incidence and mortality dropped in the 4-6-week period than the pre-lockdown period.
- Recommendations ( outside direct inference of results; but conceivable; only in body of MS) Therefore, strengthening the health system for epidemic preparedness and utilizing the lessons from past epidemics to creatively engage community entities like religious leaders, health teams, and volunteers in education, contact tracing, isolation, and burials during epidemic control might be important in controlling the EVD
