# Supplementary material for: Ebola incidence and mortality before and during a lockdown: The 2022 epidemic in Uganda
Source: PLOS Glob Public Health. 2023 Dec 22;3(12):e0002702. doi: 10.1371/journal.pgph.0002702 (PMC10745136; doi:10.1371/journal.pgph.0002702)
Supplement: Attachment — Submitted filename: Response to Reviewers.docx [file pgph.0002702.s004.docx]

**Response to Reviewer Comments**

**Reviewer #1.**

In the article titled ‘Ebola incidence and mortality before and during a lockdown: the 2022 epidemic in Uganda’, Izudi J et al assess the incidence of cases and mortality peri-lockdown during the 2022 SUDV outbreak in Uganda. I found the paper to be generally well written and the methods appropriate. I, however, hold the two concerns below:

**Response. Thank you for the valuable comments that have improved the quality of our manuscript. Please note that we have changed the nomenclature for Ebola to match the current WHO International [Statistical] Classification of Diseases [and Related Health Problems] (ICD) strongly suggesting the use of Ebola Disease (EBOD) rather than Ebola Virus Disease (Citation:** **Kuhn, J.H., Adachi, T., Adhikari, N.K.J. et al. New filovirus disease classification and nomenclature. Nat Rev Microbiol 17, 261–263 (2019).** [**https://doi.org/10.1038/s41579-019-0187-4**](https://doi.org/10.1038/s41579-019-0187-4)**).**

**Major:**

1. The authors need to inform the Uganda MOH leadership about their utilization of the data in this manner; either via the Director General or Strategic Management committee. This should easily be done by sharing a summary of the write up of results or asking for a chance to present these findings as a dissemination activity. When completed, that dissemination activity can then be noted in the paper. This will override the social controversy of use of public epidemic data in absence of awareness or permission from the owner. While it’s not unethical; this data surrounds human subjects and we need to show social responsibility to the authorities and responders.

**Response. We agree that it is important to share the findings of the study with relevant stakeholders. We have clearly described the opportunities for dissemination of the findings under the "inclusivity in global research" section attached to this manuscript as supporting information (S2). Although data reported in this study are publicly available, we have demonstrated social responsibility to the authorities and responders by presenting the findings in relevant fora in Uganda and beyond: 1) the First Annual Conference on Emerging and Re-Emerging Infectious Diseases in Africa; 2) the Infectious Diseases Institute Virtual Research Forum; and 3) the Africa Evidence Summit conference. The publication of this work is another approach to reaching the wider public and other stakeholders that are not within our reach.**

**Minor**

2. The conclusion should be restricted to inferences from the results. Any other insights should clearly be given as recommendations. As it stands now, for example, the abstracts conclusion is extended to a recommendation that is not inferred from the results of the study. I argue that any recommendation should be taken out of the abstract and left for main body of the manuscript to avoid confusion. Conclusion ‘Overall, the EVD incidence and mortality remained higher in the early weeks of the lockdown than during the pre-lockdown period nationally and in one of the two districts. With extended lockdown, incidence and mortality dropped in the 4-6-week period than the pre-lockdown period. Recommendations (outside direct inference of results; but conceivable; only in body of MS) Therefore, strengthening the health system for epidemic preparedness and utilizing the lessons from past epidemics to creatively engage community entities like religious leaders, health teams, and volunteers in education, contact tracing, isolation, and burials during epidemic control might be important in controlling the EVD.

**Response. We have restricted the conclusion to the findings and it reads: “In conclusion, the EBOD incidence and mortality remained higher in the early weeks of the lockdown than during the pre-lockdown period nationally and in one of the two districts. With extended lockdown, incidence and mortality dropped in the 4-6-week period than the pre-lockdown period.” As guided, we have deleted the previous recommendation from the abstract but maintained it within the body of the manuscript.**

**In the abstract, we added the recommendation “Therefore, reliance on known public health measures to control an EBOD outbreak is important.” This recommendation forms part of our discussion which draws lessons from the Ebola epidemic in West Africa**

**Reviewer #2:**

1. This paper would been best presented as short article. The authors also need to state whether the decrease in cases would be a natural epidemiological trend or the lock downs. restrictions imposed actually helped slow down the process.

**Response. We thank you for this comment. Our paper provides novel data regarding a lockdown to control Ebola disease for the first time in Uganda. By writing this paper as short article, much of the needed information will be missed hence a full article is most appropriate. The decision of whether this paper should have been considered as a full or short article is beyond our judgement and we leave defer it to the handling Academic Editor or the Editor-in-Chief.**

**Stating explicitly whether the decrease in cases was a natural epidemiological trend or could be attributed to the lockdowns, and whether the restrictions imposed actually slowed down the process is debatable and limiting. Our data are not able to address this question for a couple of reasons. First, we need to undertake a causal analysis to compare observed trends with the counterfactual (a measure of what the trend would have been had there been no lockdown), which is not possible given the limitations in the available data. Please note that we abstracted data from situational reports, which are published online on daily basis, often with limited variables. The details of participant characteristics as well as lockdown implementation fidelity would be crucial in undertaking a causal analysis but all these data are lacking. Second, aim of the present study was not to demonstrate impact of the lockdown but rather trends in Ebola disease before and during the lockdown period and to draw some lessons for the future epidemic response as stated in the introduction section.**

**Also, please note that we have changed the nomenclature for Ebola to match the current WHO International [Statistical] Classification of Diseases [and Related Health Problems] (ICD) strongly suggesting the use of Ebola Disease (EBOD) rather than Ebola Virus Disease (Citation:** **Kuhn, J.H., Adachi, T., Adhikari, N.K.J. et al. New filovirus disease classification and nomenclature. Nat Rev Microbiol 17, 261–263 (2019).** [**https://doi.org/10.1038/s41579-019-0187-4**](https://doi.org/10.1038/s41579-019-0187-4)**).**

2. What are the actual lessons learnt that the government of Uganda and other governments in LMICS can learn from to be able to combat epidemic.

**Response. In the discussion section titled "Lessons learned during the EBOD epidemic response and the lockdown", we have highlighted the plausible lessons learned based on the trends of EBOD before and during the lockdown. In particular, we have stated what the findings meant for future epidemic preparedness, surveillance, community engagement, financial support, diagnosis and supportive care, and the use of other proven public health prevention tools.**
